# End-of-Life Care of Acute Myeloid Leukemia Compared with Aggressive lymphoma in Patients Who Are Eligible for Intensive Chemotherapy: An Observational Study in a Japanese Community Hospital

**DOI:** 10.1089/pmr.2022.0056

**Published:** 2023-03-17

**Authors:** Masato Kokaji, Naoto Imoto, Miki Watanabe, Yutaro Suzuki, Shinji Fujiwara, Rie Ito, Toshiyasu Sakai, Satomi Yamamoto, Isamu Sugiura, Shingo Kurahashi

**Affiliations:** ^1^ Department of Postgraduate Clinical Training Center, Toyohashi Municipal Hospital, Toyohashi, Japan.; ^2^ Department of Obstetrics and Gynecology, Toyohashi Municipal Hospital, Toyohashi, Japan.; ^3^ Department of Hematology and Oncology, Toyohashi Municipal Hospital, Toyohashi, Japan.; ^4^ Department of Hematology, Nagoya Graduate School of Medicine, Nagoya, Japan.; ^5^ Department of Internal Medicine, Toyohashi Hematology Oncology Clinic, Toyohashi, Japan.

**Keywords:** acute myeloid leukemia, diffuse large B cell lymphoma, end-of-life care, palliative

## Abstract

**Background:**

Patients with hematological malignancies (HMs) are reported to receive more aggressive care at the end of life (EOL) than patients with solid tumors. However, the reasons behind this occurrence are not fully understood.

**Objectives:**

To examine whether the care at EOL for HMs is mainly because of the disease characteristics or hematologists' attitudes and systems of care, we compared the EOL care of patients with acute myeloid leukemia (AML) and diffuse large B cell lymphoma (DLBCL).

**Design:**

We retrospectively analyzed the EOL care of patients with AML and DLBCL younger than 80 years who were receiving combination chemotherapy at a city hospital in Japan.

**Results:**

Fifty-nine patients with AML and 65 with DLBCL were included. Those with AML received chemotherapy more often within their last 30 days (48% vs. 19%, *p* < 0.001) and 14 days (37% vs. 1.5%, *p* < 0.001) of life, and consulted the palliative team less frequently (5.3% vs. 29%, *p* < 0.001). In the last 3 years, the mortality rate in hematological wards decreased from 74% to 29% in the DLBCL group, but only from 95% to 90% in the AML group. In multivariate analysis, AML (odds ratio [OR] 0.065) and death before 2018 (OR, 0.077) were significant factors associated with reduced referrals to specialized palliative teams.

**Conclusion:**

Patients with AML tend to have lesser access to specialized palliative care and fewer options for their place of death than those with DLBCL. Detailed EOL care plans are needed for these patients, considering the characteristics of the disease.

## Introduction

Patients with hematological malignancies (HMs) are more likely to receive aggressive care at the end of life (EOL) and have poorer access to specialized palliative and hospice care than patients with solid tumors.^1–6^ These factors are considered suboptimal indicators of EOL,^7,8^ and in the last few years, there have been several reports on the EOL care of patients with HMs.^9^ However, these reports included patients with different HMs. Patients with HMs, such as leukemia, lymphoma, and myeloma, have very different EOL clinical presentations, and disease-specific palliative care issues need to be considered. In addition, EOL care often differs depending on the medical system in each country and the views of the department and individual physicians. To improve disease-specific EOL care, it is necessary to compare the characteristics of specific HMs in a specific geographical area.

However, to the best of our knowledge, no reports exist on the differences in EOL care between different HMs. To examine whether the highlighted EOL problems of HMs are mainly because of the disease characteristics or hematologists' attitudes to EOL and the systems of treatment, we compared the EOL care of patients with acute myeloid leukemia (AML) and diffuse large B cell lymphoma (DLBCL) in the same geographical region.

## Materials and Methods

### Patients

This was a single-center, retrospective, observational study. The study aimed to compare a group of patients who could make their own decisions about EOL care. Adult patients younger than 80 years who were diagnosed with AML and DLBCL at our institution between January 2010 and December 2019, who had received multiagent chemotherapy as first-line treatment in the hematology department, and had died from their primary disease by December 2021 were enrolled. Patients with HMs such as myelodysplastic syndrome that developed into AML were included in the AML group. Patients who were treated for DLBCL but who died of treatment-related AML were included in the AML group. Patients who died within 30 days of initial chemotherapy were excluded because we assumed that they had not experienced a good quality of EOL care.

We also excluded patients who were referred to the hematology department for HM treatment, but included patients who completed HM treatment and were then referred to a general clinic at EOL. Furthermore, patients who died from infection, hemorrhage, or unknown causes in a nonremission state were included, but those who died from apparent chemotherapy-related death, causes unrelated to HMs because they were in remission state, and unknown causes while the primary disease was in remission were excluded. Finally, patients with no recorded data for 30 days before death were excluded.

### Data collection

Patients with AML and DLBCL were selected from the XX Hospital Hematology/Oncology Database, and the eligibility was determined according to the data in their electronic medical records. Basic patient information (date of diagnosis, age, sex, family structure, and date of death), treatment details, and issues related to EOL care were collected from the electronic medical records of included patients. Issues related to EOL care were selected from indicators established by peer reviews and approved by the National Quality Forum^7,10^ or based on the results of a previous article comparing HMs and solid tumors^5^: referral rates to a specialist palliative team, hospitalization for more than 14 days within 30 days before death, death at an acute care hospital, and use of chemotherapy and molecular targeted drugs within 14 and 30 days before death. Molecular targeted drugs were defined as monoclonal antibodies and small-molecular compounds, while azacytidine was classified as chemotherapy. Chemotherapy was further divided into oral/intravenous (IV) therapy and therapy with multiple/single agents. Referral to a specialized palliative team was divided into referral to a hospice and referral to the palliative team at our hospital. In addition, we compared places of death, including the home, palliative care wards, hematology wards, other hospitals, and other locations (emergency room, intensive care unit, street); considering the changes over the years, the places of death were also compared between “after 2019” and “before 2018.” In addition, we compared the speed of disease progression at EOL, defined as the period from the date of relapse or resistance after the last administration of anticancer drugs aimed at remission to the date of death. Other parameters included a discussion of EOL wishes with the patient, rate of interventions implemented by social workers/psychological care providers in the terminal stage, and blood transfusion within 30 days before death.

### Ethical approval

This study was conducted in compliance with the Declaration of Helsinki's Ethical Guidelines for Medical Research Involving Human Subjects and was approved by the Review Board of XX Hospital. Informed consent for the collection of personal information was obtained on an opt-out basis, and details regarding the same were posted on the website of XX Hospital.

### Statistical analyses

Patient characteristics and EOL care matters for AML and DLBCL were analyzed comparatively using descriptive statistics. Fisher's exact test was used for the analysis of categorical variables. Continuous variables were analyzed using the Mann–Whitney *U* test because they did not follow a normal distribution. A multivariate analysis using logistic regression was performed to determine the factors influencing the referral rate to the palliative team. Six factors were selected as independent variables: disease, age, sex, whether the patient lived alone, whether the date of death was before 2018, and the speed of disease progression at EOL. A *p*-value of <0.05 was considered statistically significant. Values that were not evident in the medical records were considered missing values, and were not included in the analysis. All statistical analyses were performed using EZR version 1.54 (Jichi Medical University, Saitama, Japan).^11^

## Results

From January 2010 to December 2019, 684 patients (221 patients with AML and 463 patients with DLBCL) were diagnosed at our hospital, of which 113 patients with AML and 117 patients with DLBCL were aged 18 to 79 years at diagnosis and died by December 2021. In the AML group, 22 patients who had not received multiagent chemotherapy, 17 patients who died from treatment-related causes, including transplantation, one who died from other causes (pancreatic cancer), two who died from unknown causes while in remission (sudden death), nine who were referred to other hematology departments, and three who died within 30 days of initial chemotherapy were excluded.

In the DLBCL group, two patients who had not received multiagent chemotherapy, eight who died from treatment-related deaths, including treatment-related AML/myelodysplastic syndrome, 23 who died from other causes (16, solid tumors; 3, pneumonia; 1, cerebral infarction; 1, myocardial infarction; 1, heart failure; 1, Stevens–Johnson syndrome not related to hematological treatment), 10 who died from unknown causes in remission (5, sudden death; 3, referred to other general hospitals or clinics; 2, dropout), two with no information recorded in the 30 days before death, four who were referred to another hematology department, and three who died within 30 days of initial chemotherapy were excluded.

**FIG. 1.**
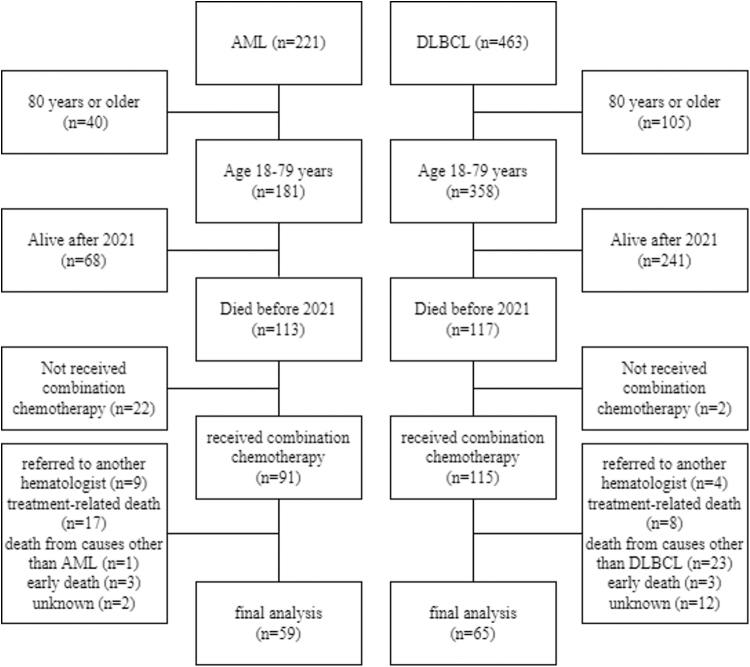
Patient selection. AML, acute myeloid leukemia; DLBCL, diffuse large B cell lymphoma.

Finally, 124 (AML group, 59; DLBCL group, 65) patients were included in the study (Fig. 1). The patients with AML were significantly younger (median 68 vs. 72 years, *p* = 0.011), but there were no significant differences in sex, living alone or not, and time from diagnosis to death between the groups. The median time from diagnosis to death was significantly shorter for the AML group (median, 9.8 months vs. 14.2 months, *p* = 0.006) (Table 1).

**Table 1.** Patients' Characteristics (*N* = 124)

	AML, ***N*** = 59 (%)^a^	DLBCL, ***N*** = 65 (%)^a^	***p***	All patients, ***N*** = 124 (%)^a^
Age in years, median (range)	68 (27–79)	72 (35–79)	0.01	70 (27–79)
Female sex	17 (29)	25 (39)	0.34	42 (34)
Live alone	3 (5.1)	9 (14)	0.13	12 (9.7)
Died after the year of 2019	20 (34)	17 (26)	0.43	37 (30)
Months between diagnosis and death, median (range)	9.8 (1.6–98.9)	14.2 (0.7–99.8)	0.01	11.3 (0.7–99.8)

^a^ Column percentage unless otherwise specified.

AML, acute myeloid leukemia; DLBCL, diffuse large B cell lymphoma.

In the last 30 days before death, patients with AML were more likely to have died in an acute care hospital (54/59 [93%] vs. 43/65 [66%], *p* < 0.001) and received chemotherapy (28/59 [48%] vs. 12/65 [19%], *p* < 0.001). Oral (19/59 [32%] vs. 0/65 [0%], *p* < 0.001) and single-agent chemotherapy (25/59 [42%] vs. 0/65 [0%], *p* < 0.001) was used more frequently, while multiagent chemotherapy (3/59 [5.1%] vs. 12/65 [19%], *p* = 0.027) was used less frequently in the AML group. There was no difference in IV chemotherapy (16/59 [27%] vs. 12/65 [19%], *p* = 0.28) and targeted therapy use (2/59 [3.4%] vs. 9/65 [14%], *p* = 0.057) between the two groups.

Both red cell (median 8 vs. 0 units, *p* < 0.001) and platelet (median 40 vs. 0 units, *p* < 0.001) transfusions were significantly more common in the AML group than in the DLBCL group. The rate of hospitalization in hematological wards for more than 14 days (37/59 [63%] vs. 45/65 [69%], *p* = 0.45) was not significantly different. In the last 14 days before death, patients with AML were more likely to receive chemotherapy (22/59 [37%] vs. 1/65 [1.5%], *p* < 0.001), but the rate of targeted therapy use was not significantly different between the groups (1/59 [1.7%] vs. 1/65 [1.5%], *p* = 1). Referral to a specialized palliative care team was significantly less common in the AML group (3/57 [5.3%] vs. 17/59 [29%], *p* < 0.001). Referral to a hospice showed a significant difference (2/59 [3.4%] vs. 15/65 [23%], *p* = 0.001).

Only one patient with AML (1.7%) and two with DLBCL (3.1%) consulted the palliative team at our hospital. Information on this aspect was unknown for one patient in the AML group and for six patients in the DLBCL group because these patients were referred to general care hospitals or general clinics after completing treatment for HMs at EOL, and it is not known whether subsequent interventions were implemented by a specialized palliative team. There were no significant differences in consultation with health care workers (17/59 [29%] vs. 25/65 [39%], *p* = 0.56), psychological care specialists (4/59 [6.8%] vs. 1/65 [1.5%], *p* = 0.19), and discussion of EOL wishes (17/59 [29%] vs. 22/65 [34%], *p* = 0.56) with the patient. Disease progression speed was also not significantly different between the two groups (Table 2).

**Table 2.** Comparison of End-of-Life Matters of Patients with Acute Myeloid Leukemia and Diffuse Large B Cell Lymphoma

	AML, ***n*** = 59 (%)^a^	DLBCL, ***n*** = 65 (%)^a^	***p***
Within last 30 days of life			
>14 days of hospitalization	37 (63)	45 (69)	0.45
Died in acute care hospital	55 (93)	43 (66)	<0.001
Chemotherapy use	28 (48)	12 (19)	<0.001
Chemotherapy use (oral)	19 (32)	0 (0.0)	<0.001
Chemotherapy use (IV)	16 (27)	12 (19)	0.28
Chemotherapy use (single agent)	25 (42)	0 (0.0)	<0.001
Chemotherapy use (combination)	3 (5.1)	12 (19)	0.027
Targeted therapy use	2 (3.4)	9 (14)	0.057
RBC transfusion median (range), unit	8 (0–42)	0 (0–14)	<0.001
PC transfusion median (range), unit	40 (0–170)	0 (0–100)	<0.001
Within last 14 days of life			
Chemotherapy use	22 (37)	1 (1.5)	<0.001
Targeted therapy use	1 (1.7)	1 (1.5)	1
Consultation with any specialized palliative care team	3 (5.3)	17 (29)	0.001
Consultation with palliative team at our hospital	1 (1.7)	2 (3.4)	1
Referral to the hospice	2 (3.4)	15 (23)	0.001
Social worker consultation about EOL	17 (29)	25 (39)	0.56
Psychological consultation about EOL	4 (6.8)	1 (1.5)	0.19
Discussion of EOL wishes with the patients	17 (29)	22 (34)	0.56
Speed of disease progression at EOL,^a^ median (range), months	1.6 (0.0–17.7)	1.6 (0.0–21.5)	0.31

^a^ Column percentage unless otherwise specified.

^b^ Defined from the day of relapse or resistance after the last administration of anticancer drugs aimed at remission to day of death.

EOL, end of life; IV, intravenous; PC, platelet concentrate; RBC, red blood cell concentrate.

**FIG. 2.**
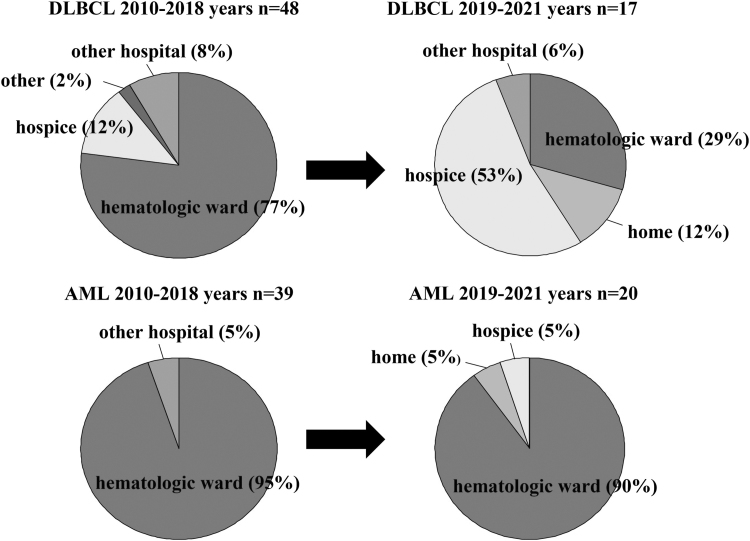
Comparison of place of death before 2018 and after 2019 in patients with AML and DLBCL.

Regarding the place of death, the AML versus DLBCL distribution was as follows: before 2018—95% versus 77% in hematology wards, 0% versus 12% at hospices, and both 0% at home; after 2019—it was 90% versus 29% in hematology wards, 5% versus 53% at hospices, and 5% versus 12% at home (Fig. 2).

Multivariate regression analysis results showed that the presence of AML (odds ratio [OR] 0.065 [95% confidence interval {CI} 0.0014–0.29], *p* < 0.001) and death before 2018 (OR 0.077 [95% CI 0.021–0.27], *p* < 0.01) were independent factors that reduced the rate of referral to a specialized palliative team (Table 3).

**Table 3.** Multivariate Logistic Regression Analysis of Consultation with Specialized Palliative Team

Patient characteristics	OR (95% CI)	***p***
AML	0.065 (0.014–0.29)	<0.001
Died before 2019	0.077 (0.021–0.27)	<0.001

Variables: disease, age, sex, whether the patient lived alone, whether the date of death was before 2019, and speed of disease at the EOL.

CI, confidence interval; OR, odds ratio.

## Discussion

We found that in the acute care ward of a Japanese community hospital, the AML group had significantly higher rates of chemotherapy use and blood transfusion in the ward within 30 days before death than the patients with DLBCL. In addition, consulting a specialized palliative care team was significantly less common among patients with AML. These differences overlapped with the differences among HMs and solid tumors that were reported in previous studies.^5^ A few previous reports have demonstrated differences in EOL issues between patients with leukemia and lymphoma.^12,13^ These population-based studies analyzed heterogeneous groups and did not specify patient-level details.

We believe that our study is unique because it focused on the details of EOL care of patients with AML and DLBCL who were eligible for intensive chemotherapy in the same health care setting. We chose AML and DLBCL because, although the characteristics of these aggressive diseases are very different, there is little difference in the environment in which they are treated: all our staff members encounter both diseases, and EOL issues related to both diseases are commonly encountered in daily clinical practice in our department. To reduce the problem of variations in EOL care due to age and general condition, only patients younger than 80 years who were eligible for combination chemotherapy were included in the study.

A short interval between the final chemotherapy treatment and death is one of the established indicators of poor-quality EOL care in patients with cancer.^7,10^ Furthermore, the benefits of hospice care at EOL are well established in cancer care.^8,14^ However, while several studies have examined EOL care using these indicators in patients with HMs in recent years,^9^ there are not enough reports on the quality of EOL care for patients with AML. Several reports have attributed the EOL problems faced by patients with HMs to the attitudes of hematology physicians.^15,16^ However, the differences between patients with AML and DLBCL observed in this report suggest that other factors, such as disease characteristics, are significant.

This report demonstrated a significant change in the place of death over the past 3 years for patients with DLBCL but not for patients with AML. The changes can be attributed to several factors, including staff awareness. A physician dedicated to palliative medicine was appointed at our hospital in April 2018, and the palliative team has been active since then. Although patients with DLBCL are rarely referred to the palliative team in our hospital, we believe that increased awareness through in-hospital educational activities has contributed to the change of place of death for patients with DLBCL.

Furthermore, our hematologic department has few members, and therefore, the impact of changing physicians may have a major impact on EOL care plans. The hospice ward to which we usually refer patients increased its capacity in March 2015. However, the increase in hospice deaths of patients with DLBCL was due to an increase in applications rather than an increase in acceptances. In Japan, in recent years, several incentives have been rolled out for acute care hospitals to shorten the patients' length of stay to ensure good patient management, and this may be a factor affecting the place of death. However, there is no direct incentive for individual physicians in this regard, and no policy changes have been made within the department to date. Therefore, staff awareness can lead to significant changes in the place of death for patients with DLBCL.

In patients with AML, on the contrary, treatments such as blood transfusions and cytoreduction were considered necessary until just before death. In addition, patients with AML may experience more rapid changes in their condition during EOL such as bleeding, infection, and bone pain.^13,17,18^ Therefore, it seems difficult to make changes only based on the awareness of the need of patients with AML. Several reports indicate the utility of interventions aimed at reducing hospitalization at EOL and increasing hospice use.^19,20^

Furthermore, several surveys on the preferred place of death, including ones from Japan, showed that more than 40% patients preferred the home and less than 20% preferred an acute care hospital.^13,21^ These reports suggest that our report of more than 90% of patients with AML dying in acute care wards is not according to their wishes, but a matter of having few options due to their particular clinical course. Thus, interventions of EOL care for patients with AML should be based on disease characteristics.

Our report also showed that patients with AML had fewer consultations with a specialized palliative care team. In our results, most consultations were referred to a hospice. There were few in-hospital consultations for both AML and DLBCL patients. This suggested that most consultations aimed to switch treatment sites because of the end of hematology treatment, rather than to consult a palliative team concurrently with hematology treatment. In these situations, the reason for the different numbers of specialized palliative team consultation is that more hematological treatments, such as transfusion^22–25^ or chemotherapy, are believed to be necessary until just before death in patients with AML. Regarding chemotherapy in EOL care, we found that only oral or single-agent chemotherapies were significantly frequent in patients with AML.

These findings indicate that patients with AML are subjected to a unique situation, where treatment is not aimed at remission but rather at cytoreduction as the best supportive EOL care.

In these circumstances, the strategies for improving the quality of EOL for patients with AML include early discussion of care goals regarding patients' EOL and provision of integrative palliative care. In our study, discussions about EOL wishes were extremely low.^13^ Furthermore, few multidisciplinary interventions were provided to patients with AML. The benefits of early EOL discussions for improving patients' QOL and increased utilization of appropriate hospice care have been reported in several studies, including studies with a Japanese cohort.^13,26–28^ The need for early specialized palliative care for patients with AML was also recently reported.^29,30^ Moreover, the integration of hematology department, hospice care wards, clinics, and inpatient palliative teams is important to facilitate palliative care for patients with AML. In addition, a system that can handle rapid changes in physical condition and provide transfusions outside the hematology department is urgently needed in the community.^9,22–25^

Finally, we note that all AML deaths occurred in hospital in the first period, while 10% of AML deaths occurred at the home/hospice in the second period. All home/hospice deaths of patients with AML occurred in 2021, the final year of this analysis. This coincides with the time period when we began discussing AML EOL problems in our department. Even in 2022, after this analysis, several AML patients younger than 80 years were still meeting their final days at home/hospice in accordance with their wishes. We emphasize the importance of discussing EOL problems of AML within the department and making a multidisciplinary effort to meet patients' wishes, while addressing the unique disease course of AML.

There are several limitations of the present study. This was a single-center cohort study. Differences of physicians and staff were not included in our analysis; however, they may have influenced the results. The reasons for the lack of integration of the palliative team and multidisciplinary professionals were not clarified. The presence of discussions on patient preferences was examined according to the data in the electronic medical records, whereas matters discussed between the medical staff and patients that were not listed in the records were not included in the analysis, and this may have led to underestimated results.

## Conclusions

In conclusion, in hospitals that refer patients to external specialized palliative care teams at the end of treatment, patients with AML are more likely to receive less access to these teams at EOL than those with DLBCL, and the former has very few options for the place of death. The matter of EOL for patients with AML may need to be discussed early on with the patients, families, and multidisciplinary professionals, taking the characteristics of the disease into consideration.

## Abbreviations Used

AML: acute myeloid leukemia
CI: confidence interval
DLBCL: diffuse large B cell lymphoma
EOL: end of life
HMs: hematological malignancies
IV: intravenous
OR: odds ratio
PC: platelet concentrate
RBC: red blood cell concentrate
